# 168 Real-world walking of people with the chronic lung disease COPD

**DOI:** 10.1093/eurpub/ckae114.184

**Published:** 2024-09-26

**Authors:** L Delgado Ortiz, J Buekers, N Chynkiamis, H Demeyer, Anja Frei, E Gimeno-Santos, C Hansen, J Hausdorff, N Hoopkinson, C P Jansen, S Koch, W Maetzler, D Megaritis, M Puhan, D Singleton, G Thoemmes, I Vogiatzis, H Watz, S Del Din, B Cauffield, C Becker, L Rochester, T Troosters, J Garcia-Aymerich

**Affiliations:** ISGlobal; Universitat Pompeu Fabra; CIBERESP, Barcelona, Spain; ISGlobal; Universitat Pompeu Fabra; CIBERESP, Barcelona, Spain; Thorax Research Foundation, Athens, Greece; Department of Rehabilitation Sciences, KULeuven; Department of Rehabilitation Sciences, Leuven, Ghent, Belgium; Epidemiology, Biostatistics and Prevention Institute, University of Zurich, Zurich, Switzerland; ISGlobal; Universitat Pompeu Fabra; CIBERESP, Barcelona, Spain; Kiel University, Kiel, Germany; Center for the Study of Movement, Cognition, and Mobility, Tel Aviv Sourasky Medical Center, Tel Aviv, Israel; Imperial College London, London, United Kingdom; Robert Bosch Gesellschaft für Medizinische Forschung, Stuttgart, Germany; ISGlobal; Universitat Pompeu Fabra; CIBERESP, Barcelona, Spain; Kiel University, Kiel, Germany; Northumbria University Newcastle, Newcastle, United Kingdom; Epidemiology, Biostatistics and Prevention Institute, University of Zurich, Zurich, Switzerland; University College Dublin, Dublin, Ireland; MerckKGaA, Darmstadt, Germany; Northumbria University Newcastle, Newcastle, United Kingdom; Pulmonary Research Institute at Lungen Clinic, Grosshansdorf, Germany; Translational and Clinical Research Institute, Faculty of Medical Sciences, Newcastle University; National Institute for Health and Care Research (NIHR), Newcastle Biomedical Research Centre (BRC), Newcastle University and the Newcastle Upon Tyne Hospitals NHS Foundation Trust, Newcastle, United Kingdom; Robert Bosch Gesellschaft für Medizinische Forschung, Stuttgart, Germany; Translational and Clinical Research Institute, Faculty of Medical Sciences, Newcastle University; National Institute for Health and Care Research (NIHR), Newcastle Biomedical Research Centre (BRC), Newcastle University and the Newcastle Upon Tyne Hospitals NHS Foundation Trust, Newcastle, United Kingdom; Department of Rehabilitation Sciences, KULeuven, Leuven, Belgium; ISGlobal; Universitat Pompeu Fabra; CIBERESP, Barcelona, Spain

## Abstract

**Purpose:**

Walking is crucial for active and healthy ageing, but it changes with age and in the presence of diverse health conditions, such as non-communicable diseases. In the field of the chronic lung disease COPD (chronic obstructive pulmonary disease), extensive research has been done on physical activity limitation, but little is known about the patterns of walking during daily life (i.e., real-world). We aimed to assess the levels and distribution of real-world walking (walking activity and gait parameters) in people with COPD compared with healthy adults, and across disease severity; and to identify domains of walking in COPD, using a single wearable device.

**Methods:**

In a cross-sectional study, 550 people with COPD (37% women, with a mean (SD) age of 68 (8) years, postbronchodilator FEV1 54 (21) %) and 19 age-matched healthy adults were recruited in ten cities across eight countries. Subjects were given a single wearable device to wear at their lower back for one week. Twenty-five walking activity and gait parameters (e.g., number of steps, number of walking bouts (WB)>30s, walking speed in WB > 30s, cadence in WB > 30s, walking speed and cadence variability) were derived from the device outputs.

**Results:**

Most walking activity and gait parameters were reduced in people with COPD compared to healthy adults, and according to COPD severity. Differences remained statistically significant after adjusting for age, sex, height and comorbidities in multivariable analyses. Factor analysis identified six distinct walking domains: Amount of walking, Patterns of walking, Pace, Rhythm, Pace variability and Rhythm variability.

**Conclusion:**

Real-world walking in people with COPD is a multi-dimensional behaviour that is impaired as the disease progresses. These results may be used to set priorities and improve patient centricity in clinical practice, research and public health initiatives.

**Support/Funding Source:**

This work was supported by the Mobilise-D project that has received funding from the Innovative Medicines Initiative 2 Joint Undertaking (JU) under grant agreement No. 820820. This JU receives support from the European Union’s Horizon 2020 research and innovation programme and the European Federation of Pharmaceutical Industries and Associations (EFPIA).

